# Using the Scaffold of FDA-Approved Drugs with Trypanocidal Activity to Identify New Anti-*Trypanosoma cruzi* Agents: An In Silico and In Vitro Approach

**DOI:** 10.3390/molecules31081327

**Published:** 2026-04-17

**Authors:** Lenci K. Vázquez-Jiménez, Alonzo González-González, Timoteo Delgado-Maldonado, Rogelio Gómez-Escobedo, Guadalupe Avalos-Navarro, Adriana Moreno-Rodríguez, Alma D. Paz-González, Eyra Ortiz-Pérez, Benjamín Nogueda-Torres, Gildardo Rivera

**Affiliations:** 1 Laboratorio de Biotecnología Farmacéutica, Centro de Biotecnología Genómica, Instituto Politécnico Nacional, Reynosa 88710, Mexico; lenka.18@hotmail.com (L.K.V.-J.); al.gonzalez.gonzalez88@gmail.com (A.G.-G.); titi_999@live.com (T.D.-M.); apazg@ipn.mx (A.D.P.-G.); eortizp@ipn.mx (E.O.-P.); 2 Secretaría de Ciencia, Humanidades, Tecnología e Innovación (SECIHTI), Ciudad de México 03940, Mexico; 3 Escuela Nacional de Ciencias Biológicas, Instituto Politécnico Nacional, Ciudad de México 11340, Mexico; rogelio.gomez14@gmail.com; 4 Departamento de Ciencias Médicas y de la Vida, Centro Universitario de la Ciénega (CUCIÉNEGA), Universidad de Guadalajara, Av. Universidad 1115, Lindavista, Ocotlán 47820, Mexico; guadalupe.avalos5337@academicos.udg.mx; 5 Laboratorio de Estudios Epidemiológicos, Clínicos, Diseños Experimentales e Investigación, Facultad de Ciencias Químicas, Universidad Autónoma “Benito Juárez” de Oaxaca, Avenida Universidad S/N, Ex Hacienda Cinco Señores, Oaxaca 68120, Mexico; arimor10@hotmail.com

**Keywords:** *Trypanosoma cruzi*, *Trans*-sialidase, triosephosphate isomerase, drug repositioning, FDA drugs, molecular docking

## Abstract

Chagas disease affects millions of people worldwide, including those in Latin America. The only drugs available for its treatment are benznidazole and nifurtimox. However, these drugs present high toxicity and limited efficacy. Therefore, the search for new treatments continues. In this regard, computer-assisted drug design has been implemented in scientific research for drug repurposing, allowing for reduced costs and time. Therefore, the objective of this work was to search for analogs of FDA-approved drugs with activity against *Trypanosoma cruzi* through ligand-based virtual screening and their biological evaluation against blood trypomastigotes. The compound **TD-095** (LC_50_ = 48.60 and 13.75 µM), a ketanserin analogue, **TS-936** (LC_50_ = 71.55 and 37.54 µM), a terfenadine analogue, and **TD-831** (LC_50_ = 75.94 and 26.17 µM), a sulfasalazine analogue, were considered as potential *trans*-sialidase inhibitors; **TIM-967** (LC_50_ = 69.70 and 39.69 µM) and **LK-284** (LC_50_ = 116.7 and 82.29 µM), two sulfonylurea analogues, were considered as potential triosephosphate isomerase inhibitors, showing better trypanocidal activity against NINOA and INC-5 strains, respectively, than the reference drugs. Molecular dynamics simulations predicted the stability of the compounds in complex with their respective proteins. Finally, the ADMET predictive analysis showed favorable properties for the compounds. These results support continued research into new agents against *Trypanosoma cruzi*, using structures of drugs already approved by the FDA.

## 1. Introduction

*Trypanosoma cruzi* (*T. cruzi*) is responsible for Chagas disease, a major public health problem in Latin America, with approximately eight million people infected worldwide [1]. In Mexico, approximately one million infected people have been reported, with nearly 8000 new cases per year [2].

Even today, only two drugs (nifurtimox and benznidazole) exist for the treatment of Chagas disease. These cause severe adverse effects, leading to discontinuation of treatment by the patient, such as dermatitis, peripheral neuropathy, anorexia and weight loss, nausea, vomiting, insomnia, bone marrow suppression, abdominal pain, headache, and dizziness or vertigo [3,4]. Therefore, there is a need to continue developing new therapeutic options. In this context, the *trans*-sialidase (TS) and triosephosphate isomerase (TIM) proteins of *T. cruzi* have been considered as potential drug targets [5,6]. It has been described that the TS enzyme of *T. cruzi* (*Tc*TS) is crucial for the survival of this parasite and the establishment of an effective infection [7], while the TIM enzyme of *T. cruzi* (*Tc*TIM) catalyzes the isomerization of glyceraldehyde-3-phosphate and dihydroxyacetone phosphate in the glycolytic pathway, an essential mechanism for the generation of net energy in the form of ATP in *T. cruzi* [8].

**Figure 1:**
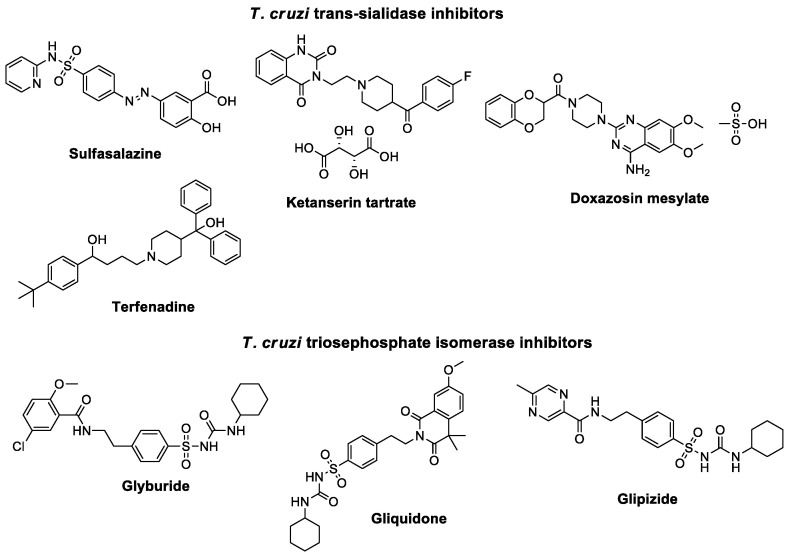
Chemical structures of FDA-approved drugs with activity against *T. cruzi* targeting TS and TIM proteins.

On the other hand, computer-aided drug design (CADD) is a method that enables the development of new compounds in a shorter timeframe and increases the likelihood of discovering new drugs [9]. In this regard, using CADD strategies and validating the results in biological models, our research group successfully repurposed seven existing drugs; (ketanserin tartrate, terfenadine, sulfasalazine, doxazosin mesylate as potential inhibitors of *Tc*TS, and glipizide, glibenmide, and gliclazide as potential inhibitors of *Tc*TIM for the potential treatment of Chagas disease (Figure 1) [10,11]. Therefore, in this study, the chemical structures of these drugs were used to identify structurally similar compounds through ligand-based virtual screening and molecular docking refinement. This allowed us to select the best candidates for in vitro evaluation against two strains of *T. cruzi*, aiming to identify molecules with enhanced biological activity. Furthermore, the most promising compounds were subjected to molecular dynamics simulations and ADMET analysis to predict their stability when bound to the target protein and their pharmacokinetic properties, which could lead to a new therapeutic option for Chagas disease.

## 2. Results

### 2.1. Protein–Ligand Analysis

Initially, a molecular docking analysis was performed on the previously used proteins *Tc*TS and *Tc*TIM, which were obtained from the Protein Data Bank (PDB) database, along with their corresponding co-crystallized ligands, 3-deoxy-2,3-dehydro-*N*-acetylneuraminic acid (DANA) and 3-(2-benzothiazolylthio)-1-propanesulfonic acid (BTS), respectively [10,11]. The binding free energy (BFE) of each ligand, as well as the interaction profile with the amino acid residues on the active site, is shown in Table 1.

### 2.2. Ligand-Based Virtual Screening from MolPort Database

Considering seven FDA-approved drugs as a scaffold, a virtual screening of the MolPort database was performed to obtain the analogous compounds by 50 % structural similarity. After that, Lipinski and Veber rules were applied, and finally, a molecular docking analysis was performed on each respective molecular target. Subsequently, the compounds that had better BFE than the control ligand were selected (Table 1). Then, all those compounds that shared at least one interaction with the control ligand were selected and grouped. Table 2 shows the number of compounds obtained by applying each inclusion criterion. Glipizide, glyburide, and gliquidone scaffolds showed the same 10,000 compounds with structural similarity. Eight compounds were ultimately selected for acquisition based on their commercial availability. Six *Tc*TS compounds and two *Tc*TIM compounds were selected and evaluated against *T. cruzi* blood trypomastigotes.

### 2.3. Molecular Docking Analyses

**Figure 2:**
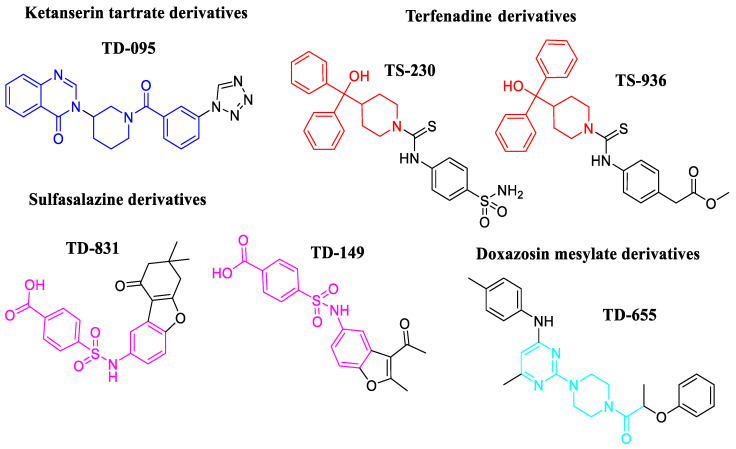
Chemical structures of the selected compounds as potential *Tc*TS inhibitors. The blue color highlights the ketanserin scaffold, the red indicates the terfenadine scaffold, and sulfasalazine and doxazosin are shown in pink and cyan, respectively.

The six compounds (Figure 2) selected as potential *Tc*TS inhibitors for biological evaluations against *T. cruzi* trypomastigotes were analyzed by their BFE and interaction profile.

**Figure 3:**
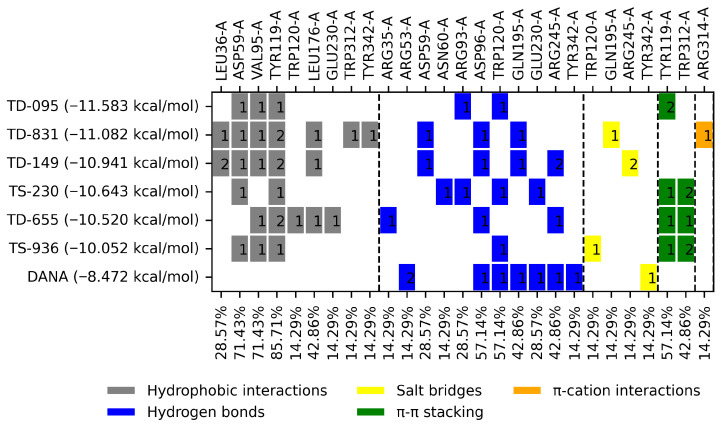
Interaction profile of the six compounds selected as potential *Tc*TS inhibitors. The number displayed inside the interaction box represents the number of interactions.

The selected compounds had BFE values ranging from −11.58 to −10.94 kcal/mol, while the control ligand (DANA) had a BFE of −8.47 kcal/mol (Figure 3). Compound TD-095 had the best BFE value (−11.58 kcal/mol) and eleven interactions, while compound TD-149 (−10.94 kcal/mol) was the compound that presented the highest number of interactions (fourteen), followed by compound TD-831 (thirteen).

**Figure 4:**
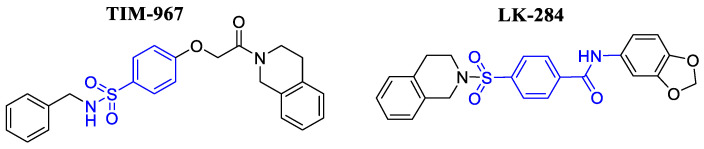
Chemical structures of the compounds selected as potential *Tc*TIM inhibitors.

**Figure 5:**
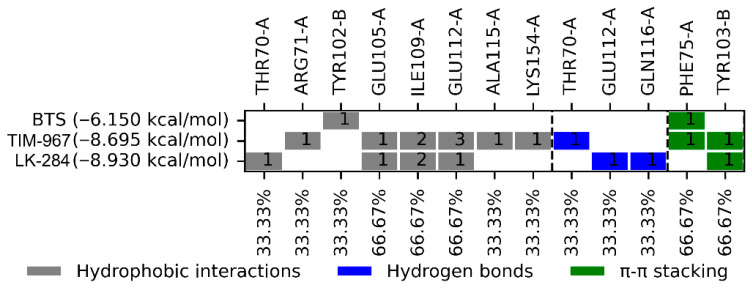
Interaction profile of the two compounds selected as potential *Tc*TIM inhibitors. The number displayed inside the interaction box represents the number of interactions.

Finally, the chemical structures of compounds TIM-967 and LK-284 selected as potential *Tc*TIM inhibitors, are presented in Figure 4. The interactions and BFE of compounds TIM-967 and LK-284 that were commercially purchased for in vitro evaluations against *T. cruzi* are depicted in Figure 5.

Compounds TIM-967 and LK-284 had docking scores of −8.69 and −8.93 kcal/mol, respectively. TIM-967 and LK-284 had hydrophobic, hydrogen bonding, and π-π stacking interactions. On the other hand, the ligand co-crystallized with the protein (BTS) showed a docking score of −6.15 kcal/mol and only two interactions (TYR102-B and PHE75-A).

### 2.4. Trypanocidal Activity

The eight selected compounds were evaluated against the trypomastigote form of the NINOA and INC-5 strains of *T. cruzi* (Table 3). Four compounds (TD-095, TS-936, TD-831, and TD-149) selected through virtual screening as potential *Tc*TS inhibitors had better activity than the two reference drugs against the NINOA strain, with LC_50_ values between 40.54 and 75.94 µM). On the other hand, compounds TD-095, TS-936, TS-230, and TD-831 exhibited better activity values than the reference drugs against the INC-5 strain (Table 3). Furthermore, the compounds (TIM-967 and LK-284) selected as potential *Tc*TIM inhibitors in the in vitro evaluation against *T. cruzi* trypomastigotes showed better activity than the reference drugs against both strains (Table 3).

### 2.5. Statistical Analysis

Statistical analysis (ANOVA, *p* < 0.05) showed significant differences between the compounds evaluated against *T. cruzi*. In general, several compounds showed better LC_50_ values than the reference drugs. Among the compounds targeting *Tc*TS, TD-095 highlights one of the lowest LC_50_ values against the NINOA and INC-5 strains, although TD-831 and TS-936 also showed relevant biological activity.

On the other hand, derivatives associated with *Tc*TIM, such as TIM-967, showed better activity than benznidazole and nifurtimox. The superscript letters (a–d) confirm the statistical differences between means according to Tukey’s test, allowing the identification of compounds with better antiparasitic performance.

### 2.6. Molecular Dynamics Analyses

#### 2.6.1. TcTS Complexes

**Figure 6:**
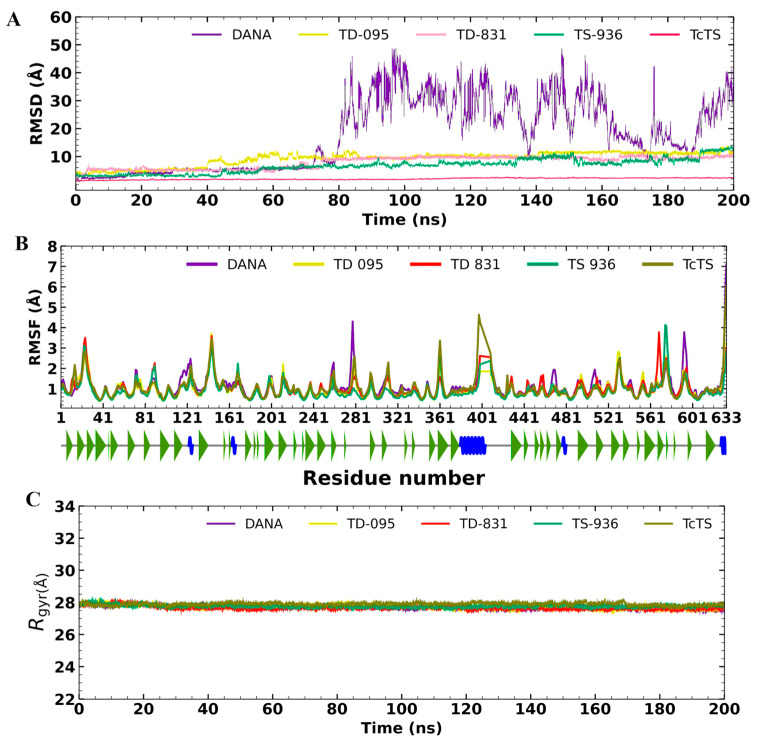
Global analysis of MD simulations of *Tc*TS in complex with the potential inhibitors. (**A**) RMSD plot, (**B**) RMSF plot, and (**C**) Rgyr plot. In the RMSF graph at the bottom, beta strands are represented in green and alpha helices in blue.

Molecular dynamics (MD) simulation was performed to predict the stability of *Tc*TS in complex with the potential inhibitors. The analysis was carried out in triplicate, yielding an average root mean square deviation (RMSD) for *Tc*TS of 1.98 ± 0.17 Å with a coefficient variation (CV) of 8.86 % throughout the MD simulation (Figure 6A). The reference inhibitor DANA showed an average RMSD of 17.82 ± 17.32 with a CV of 97.20 %, and stability was observed during the first 40 ns (RMSD = 5.43 ± 2.17 Å), while the ligand TD-095 had an average RMSD of 9.24 ± 1.81 Å with a CV of 19.58 %. On the other hand, the ligand TD-831 in complex with *Tc*TS showed the best behavior, with an average RMSD of 6.68 ± 3.36 Å and a CV of 50.40 %. The ligand TS-936 exhibited minimal fluctuations during the MD simulation, with an average global RMSD of 7.94 ± 2.99 Å (CV of 37.76 %).

The root-mean square fluctuation (RMSF) analysis of all ligands during the MD simulation with *Tc*TS showed minimal fluctuations (Figure 6B). Only the ligands TD-836, TS-936, and the DANA inhibitor showed slight changes in regions containing loop-forming residues in the protein structure, which explains these fluctuations. Therefore, the fluctuations observed during the receptor-ligand interaction in the MD studies were acceptable. Figure 6C shows the radius of gyration (Rgyr) of free *Tc*TS (apoprotein) and its behavior in complex with each ligand. The average Rgyr value for the apoprotein was 23.92 ± 0.12 Å. In all cases, the holo-protein in complex with each ligand showed an average Rgyr value of 23.76 ± 0.16 Å.

#### 2.6.2. *Tc*TIM Complexes

**Figure 7:**
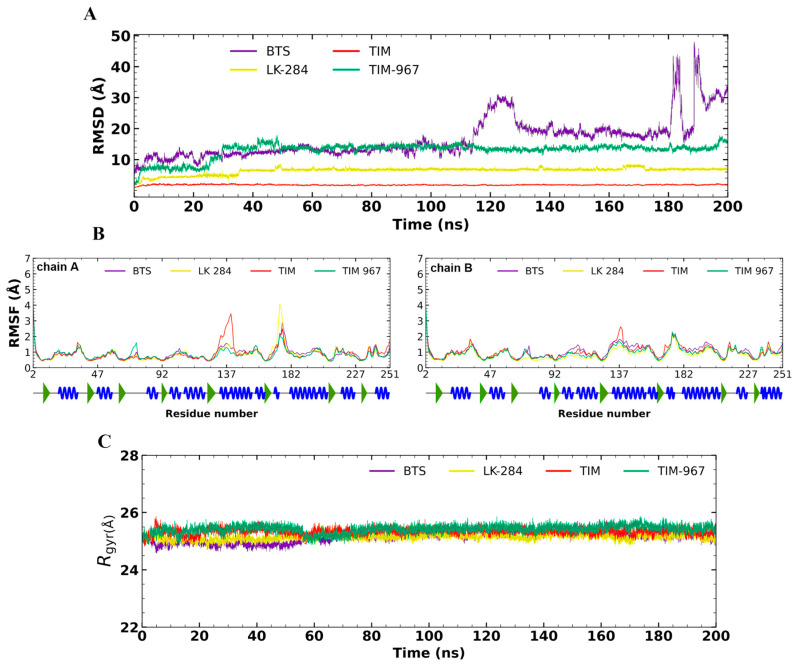
**Global** analysis of MD simulations of *Tc*TS in complex with the potential inhibitors. (**A**) RMSD plot, (**B**) RMSF plot, and (**C**) Rgyr plot. In the RMSF graph at the bottom, beta strands are represented in green and alpha helices in blue.

Triplicate MD simulation analysis of the apo-form of *Tc*TIM predicted an average RMSD of 1.98 ± 0.17 Å and a CV of 9.87 % (Figure 7A). On the other hand, the inhibitor BTS showed a high RMSD value of 16.6 ± 5.35 Å and a CV of 32.13 %. The ligands TIM-967 and LK-284 exhibited similar behavior, requiring the first 35 ns of the simulation to properly position themselves at the *Tc*TIM interface and bind tightly, ultimately achieving acceptable stability by the end of the MD simulation (RMSD = 12.91 ± 0.93 and 6.39 ± 1.05 Å, with CVs of 7.20 and 16.51 %, respectively). RMSF calculations for *Tc*TIM and the ligands during the MD simulation did not show significant fluctuations (Figure 7B).

Figure 7C shows the Rgyr values obtained for *Tc*TIM, both in its free form (apoprotein) and in complex with each of the evaluated ligands. The average Rgyr value for the apo-protein was 21.71 ± 0.13 Å. For the ligands, the average Rgyr values ranged between 21.60 ± 0.16 Å and 21.56 ± 0.12 Å.

### 2.7. Molecular Docking on Human Triose Phosphate Isomerase

**Figure 8:**
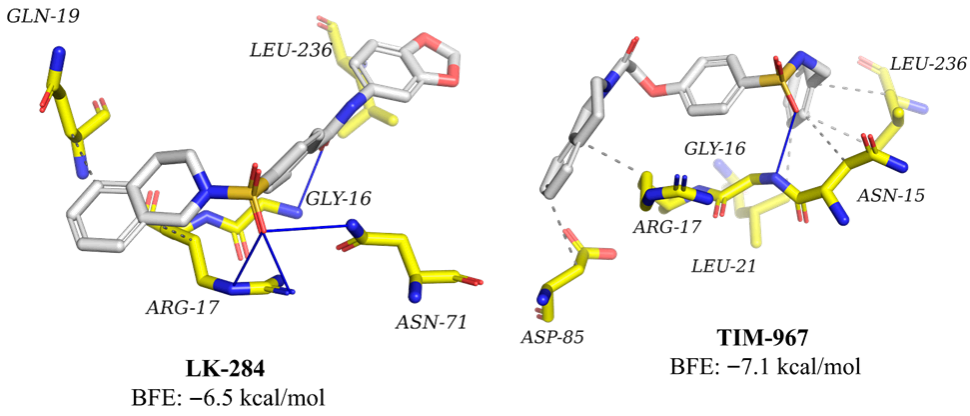
Interaction profile of compounds LK-284 and TIM-967 with *Hs*TIM residues. Hydrogen bonds are represented as blue intense lines, and hydrophobic interactions as a grey dashed line.

The compounds LK-284 and TIM-967, selected as the two most promising potential inhibitors of *Tc*TIM, were evaluated using molecular docking at the *Homo sapiens* triosephosphate isomerase (*Hs*TIM) interface (Figure 8) to predict their potential as selective trypanocidal agents. These ligands exhibited different conformations within the protein region, with BFE values of −6.5 and −7.1 kcal/mol, respectively. The main interactions observed were hydrophobic contacts and hydrogen bonds with amino acid residues different from those present in the protozoan protein (Figure 5).

### 2.8. In Silico Pharmacokinetic Analysis of Isomerase

The physicochemical properties of the best trypanocidal compounds (TD-095, TS-936, TD-831, TIM-967, and LK-284) were predicted using the ADMETlab 3.0 website (Table 4). According to the results, all compounds comply with Lipinski’s rules [12]. Regarding solubility (LogS), the values obtained ranged from −2.97 to −5.28 log mol/L, suggesting moderate to low solubility, with LK-284 being the compound with the lowest predicted solubility. Interaction analysis with cytochrome P450 isoforms (1A2, 2C19, 2C9, and 2D6), key enzymes in drug metabolism [13], showed that TD-095 did not exhibit inhibitory activity against any of the evaluated isoforms. In contrast, TD-831 did not show inhibitory effects on any of the analyzed isoforms, while compounds TS-936, TIM-967, and LK-284 inhibited two or three isoforms. These results suggest possible differential metabolic interactions between the compounds.

The distribution of the compound into different body compartments was predicted using P-glycoprotein (P-gp) efflux and blood–brain barrier (BBB) penetration parameters [13,14,15]. All compounds were predicted to be potential substrates of P-gp. Although compounds TD-095, TS-936, and TD-831 do not penetrate the BBB, hepatotoxicity is one of the main reasons for drug withdrawal from the market [15]. The predicted hepatotoxicity analysis indicated that TD-831 and LK-284 could pose a hepatotoxic risk, while TD-095, TS-936, and TIM-967 were classified as inactive for this parameter. Finally, the estimated median lethal dose (LD_50_) (in mg/kg body weight), a measure of acute toxicity, with values ranging from 650 to 4000 mg/kg (Table 5).

In the case of the reference drugs, benznidazole and nifurtimox, both meet the criteria of Lipinski’s rule, exhibiting low molecular weights (260.09 and 287.06 g/mol, respectively), adequate TPSA and LogP values, suggesting good oral absorption. The LogS values (−2.86 and −3.15, respectively) indicate moderate solubility. Regarding permeability, benznidazole showed the ability to cross the BBB, while nifurtimox did not. Both were predicted to be substrates of P-gp and did not show relevant inhibition of the evaluated CYP isoforms, except for benznidazole, which showed possible inhibition of CYP2C19. Furthermore, neither exhibited the predicted hepatotoxicity, and their estimated LD_50_ values (2000 and 1500 mg/kg, respectively) reflect moderate acute toxicity.

## 3. Discussion

### 3.1. Protein–Ligand Analysis

The DANA ligand had a docking score of −8.1 kcal/mol and hydrogen bond, salt bridge, and water bridge type interactions on the active site of *Tc*TS (Table 1). The interactions found for the *Tc*TS-DANA complex coincide with those previously reported as ARG245 and ARG314, part of the catalytic triad, and the amino acid residues TYR342 and GLU230 that stabilize the transition state reaction species, among other residues ARG53, ASP96, TRP120, and TYR119 [16,17].

Finally, the BTS ligand had a binding score of −6.2 kcal/mol over *Tc*TIM with hydrogen bond, hydrophobic, and π-stacking interactions with TYR103, TYR102, ILE69, and PHE75 at the *Tc*TIM interface (Table 1), which have also been previously described in the crystal structure and by our working group [18,19].

### 3.2. Molecular Docking Analyses

Once the virtual screening and molecular docking analysis were completed, the interaction profiles of the control ligands and the selected compounds for the biological assays were analyzed (Figure 3 and Figure 5), revealing that the predominant hydrophobic interaction for the six potential *Tc*TS inhibitors occurred with the residue TYR119 in 85.71% of cases, followed by a π-π interaction with the same residue. This suggests that the phenyl tyrosine residue is key to accommodating inhibitors on the active site of *Tc*TS. Therefore, future anti-Chagas drug design studies may target the TYR119 residue. On the other hand, the reference inhibitor DANA exhibited an acceptable BFE value. Furthermore, DANA interacted with the residues ARG53, ASP96, TRP120, GLN195, GLU230, ARG245, and TYR342 through hydrophobic, π-π-stacking, hydrogen bonds, ionic and π-cation interactions. Compounds that showed greatest number of interactions than DANA were TD-149 (ASP96, GLN195, and ARG245), followed by TD-655 (ASP96 and ARG245), TD-831 (ASP96 and GLN195), and TS-230 (TRP120 and GLU230).

On the other hand, the ligand co-crystallized with the *TcTIM* protein (BTS) showed a docking score of −6.15 kcal/mol and only two interactions (TYR102-B and PHE75-A), the same ones described by Téllez-Valencia et al. [18]. Of the two interactions shown by the BTS ligand, only one is common with TIM-967 and LK-284: the PHE75-A interaction via π-π stacking. Furthermore, it was observed that TIM-967 and LK-284 exhibit common interactions with GLU105-A, ILE109-A, GLU112-A, and TYR103-B, which are predicted to be important for compound binding in the protein [18,19]. These findings suggest that these compounds could bind to the *Tc*TIM interface and disrupt it, leading to lethality in the parasite.

### 3.3. Trypanocidal Activity

All compounds commercially acquired were assessed to evaluate their trypanocidal activity on trypomastigotes of NINOA and INC-5 strains. The initial screening was at 50 μg/mL, and those compounds that showed inhibition of parasite proliferation were subjected to LC_50_ calculation. Our results showed that the compounds identified as *Tc*TS inhibitors (TD-095, TS-936, TD-831, and TD-149) had LC_50_ values between 40.54 and 75.94 μM. These findings demonstrate that these new compounds have a more potent effect than the reference drugs Nfx and Bzn. Meanwhile, these compounds showed comparable activity to that reported for ketanserin, terfenadine, sulfasalazine, and doxazosin in both *T. cruzi* strains [10]. Thus, the trypanocidal activity is attributed to TS inhibition. Further enzymatic assessment is required to validate this idea. On the other hand, similar findings were observed with compounds as potential *Tc*TIM inhibitors (TIM-967 and LK-284) against *T. cruzi* trypomastigotes in both strains. Lastly, the data presented here demonstrates that searching for FDA-approved drug analogs with antiparasitic activity is a promising strategy for identifying new trypanocidal agents targeting proteins exclusive to *T. cruzi*.

Furthermore, a preliminary structure-activity relationship analysis reveals differences between the scaffolds evaluated against both parasite strains. Within the group of analogs targeting *Tc*TS, the ketanserin-based derivative TD-095 showed the best overall activity, particularly against the INC-5 strain (LC_50_ = 13.75 µM), suggesting that this scaffold promotes productive interactions with the catalytic site. In contrast, the doxazosin analog (TD-655) was inactive against both strains, indicating that the loss of aromatic groups and/or hydrophobic regions could affect trypanocidal activity. The terfenadine derivatives exhibited intermediate behavior; TS-936 maintained moderate activity against both strains, while TS-230 only showed an effect against the INC-5 strain, suggesting that small variations in substituents impact potency. Similarly, sulfasalazine analogs exhibited a strain-dependent profile, with TD-831 maintaining moderate activity against both strains, while TD-149 was selective for NINOA, demonstrating that the arrangement of polar groups (such as the carboxylate) influences biological selectivity. On the other hand, the two compounds selected as potential inhibitors of *Tc*TIM showed activity, with TIM-967 exhibiting the best LC_50_ values. This suggests that the sulfonylurea core, although compatible with the protein interface, could be further optimized.

In general, trypanocidal activity is favored when the hydrophobic aromatic scaffold is combined with one or two strategically positioned polar groups, primarily amines and sulfonamides, capable of forming hydrogen bonds and electrostatic interactions with the target.

### 3.4. Molecular Dynamics Analyses

#### 3.4.1. TcTS Complexes

The stability of the compounds that exhibited the best trypanocidal activity (TD-095, TD-831, and TS-936) was predicted using MD simulations in complex with *Tc*TS (Figure 6). The apo-protein structure analyzed showed stability (RMSD < 2 Å). The reference inhibitor DANA also showed stability during the first 45 ns; however, the greatest fluctuations were observed between 76 ns and the end of the simulation (Figure 6A). This behavior suggests that DANA adopts a different conformation within the protein binding site compared to its initial position. However, this new pose is not strong enough to reach the desired stability through MD simulation.

In terms of RMSD, all three *Tc*TS inhibitors demonstrated better stability than the co-crystallized ligand DANA; these findings support that the interactions predicted by molecular docking are sufficiently strong to observe protein–ligand complex stability under the conditions tested. The RMSF analysis of the ligands TD-095, TD-831, and TS-936 bound to *Tc*TS showed minimal fluctuations in the regions containing residues that form loops in the protein structure (Figure 6B). In addition, the Rgyr analysis results indicate that both the apo-protein and the holo-protein remain stable throughout the MD simulation at 200 ns, and no significant differences were observed, suggesting that the protein remains compact in the simulation.

#### 3.4.2. *Tc*TIM Complexes

The MD simulation analysis of the apo form of *Tc*TIM revealed stability (RMSD < 2 Å). The reference inhibitor, BTS, showed significant fluctuations during the simulation, consistent with its high RMSD value. These changes suggest that the interactions predicted in the docking studies are not strong enough to maintain stability during the MD simulation.

In general, the ligands TIM-967 and LK-284 in complex with *Tc*TS displayed fluctuations in the first 35 ns. After this time, the observed fluctuations were minimal (RMSD < 5 Å), particularly for compound LK-284, which achieved the greatest stability throughout the simulation. Thus, this result highlights that the novel *Tc*TIM inhibitors discovered here can bind to the protein interface and remain stable. This implies that the *Tc*TIM function is compromised and becomes lethal in *T. cruzi*, causing trypanocidal activity in trypomastigotes. The RMSF analysis shows that the apo form of the TIM protein has a pronounced loop in the region of residues 130–140 (chain A). However, when the protein forms a complex with the inhibitors, this loop is reduced. These findings suggest that the presence of small molecules helps stabilize the fluctuations of these residues in chain A. Furthermore, the ligand LK-284 causes a slight expansion of the protein loop in the region of residues 170–180. The rest of the protein structure remains unchanged. Based on the Rgyr results, it can be inferred that both the apo and holo forms of the *Tc*TIM protein remain stable during the 200 ns MD simulation, without significant changes.

### 3.5. Molecular Dynamics on Human Triose Phosphate Isomerase

To predict the selectivity of the most promising potential *Tc*TIM inhibitors (LK-284 and TIM-967), molecular docking simulations were performed at the *Hs*TIM interface. Both compounds showed lower affinity for the *Hs*TIM interface (−6.5 and −7.1 kcal/mol, respectively) than that predicted for *Tc*TIM (−8.93 and −8.69 kcal/mol, respectively) (Figure 8). Furthermore, they exhibited a lower number of interactions with *Hs*TIM (four and six interactions, respectively) compared to those with the *Tc*TIM protein (eight and twelve interactions, respectively). This could be since the interfaces of *Tc*TIM and *Hs*TIM have a 48% sequence difference, making the interface a key binding site for these compounds [19,20].

### 3.6. In Silico Pharmacokinetic Analysis

In general, the physicochemical and ADMET properties of the best trypanocidal compounds (TD-095, TS-936, TD-831, TIM-967, and LK-284) were shown to satisfy Lipinski’s rule criteria for small molecules (MW < 500, RB < 10, HBA < 10, HBD < 5, Log P < 5). These data are consistent with in vitro results, which show good solubility profiles. This also indicates that they are likely membrane-permeable and readily absorbed by passive diffusion in the human intestine [21]. All compounds exhibited TPSA values < 140 Å^2^ and LogP < 5, indicating a good probability of oral absorption. All compounds were shown to be potential substrates of P-gp, and therefore, their bioavailability could be affected. Meanwhile, predictions for TIM-967 and LK-284 showed BBB penetration. TD-095, TS-936, and LK-284 do not exhibit BBB permeability, which may be advantageous if central nervous system (CNS) effects are to be avoided. Predicted for CYP450 inhibition showed that TS-936, TD-831, TIM-967, and LK-284 are active in inhibiting some of the isoforms. Therefore, in vitro studies on these enzymes are needed to corroborate predictions. All compounds are P-gp substrates, which could limit their brain accumulation and bioavailability; however, this may also reduce CNS toxicity. Compounds TD-095, TS-936, and TIM-967 were not predicted to be hepatotoxic agents, suggesting a low risk of liver damage in the early stages of drug development. Finally, the predicted LD_50_ ranged from 650 to 4000 mg/kg, indicating low to moderate acute toxicity. TIM-967 showed the lowest value (650 mg/kg), suggesting greater relative toxicity, while TD-831 and LK-284 showed the highest values (4000 mg/kg), indicating a better safety margin. Compound TD-095, with its desirable ADMET profile, stands out as a promising candidate for future studies as an anti-*T. cruzi* agent targeting the TS protein.

Compared to benznidazole and nifurtimox, the analyzed compounds exhibit physicochemical profiles consistent with Lipinski’s rule and similar absorption parameters, although with greater lipophilicity and, in some cases, lower solubility. Unlike the reference drugs, which show minimal inhibition of CYP isoforms, several of the new compounds inhibit two or more enzymes, which could imply a greater risk of metabolic interactions. Regarding distribution and toxicity, some compounds share the absence of predicted hepatotoxicity and comparable LD_50_ values, although greater toxicological variability is observed among them.

Taken together, the in silico and experimental results show a correlation between the observed trypanocidal activity and the molecular properties of the evaluated compounds. Molecular docking studies demonstrated adequate binding affinity for *Tc*TS and *Tc*TIM, highlighting key interactions with previously described catalytic and interface residues, supporting a mechanism of action targeting essential and unique targets of *T. cruzi*. These interactions resulted in stable protein–ligand complexes during molecular dynamics simulations, where the compounds showed less fluctuation and greater stability than the reference ligands, suggesting robust and functional binding under dynamic conditions.

Furthermore, selectivity analyses revealed a lower affinity for human TIM, indicating a potentially favorable selective profile that may reduce the risk of off-target effects. Finally, ADMET predictions support the pharmacological viability of the compounds, showing good stability, suitable physicochemical properties, and low in silico toxicity. However, the predictions would need to be confirmed in silico through in vitro analysis to position these compounds as promising candidates for the development of new anti-*T. cruzi* agents.

## 4. Materials and Methods

### 4.1. Protein Preparation

The crystal structures of *Tc*TIM, *Tc*TS, and *Hs*TIM were recovered from the PDB database with accession codes 1SUX, 1MS8, and 4POC, respectively. All crystal structures were prepared using the open-access software UCSF-Chimera 1.15 [22], removing all co-crystallized molecules (including H_2_O molecules) and using the built-in Dock Prep tool to add hydrogen atoms and Kollmann partial charges to the protein crystals. In addition, the protonation of ionizable residues (His, Asp, Glu, Lys, Arg) was verified at physiological pH. The prepared protein was saved in pdb format.

### 4.2. Ligand Library Preparation

Eleven ligand libraries were obtained from the MolPort chemical database (https://www.molport.com/shop/index) (accessed on 20 February 2025). To ensure moderate structural similarity that allows for chemical diversity, a fingerprint Tanimoto similarity was chosen at 0.5 with each of the repurposed parent ligands: ketanserin, terfenadine, sulfasalazine, doxazosin, glipizide, gliquidone, and glyburide. The search was limited to commercially available compounds in the database. Each exported sdf file was converted to smi format, and it was then cleaned of any salts or additional molecules included in the initial sdf file but not part of the main ligand. Then, the cleaned smi file was imported into and filtered out using DataWarrior software, considering Lipinski’s (MW ≤ 500 g/mol, RB < 10, HBA < 10, HBD < 5, and Log P < 5) and Veber (TPSA ≤ 140 Å^2^) rules as inclusion criteria for selected compounds, compounds were exported in a 2D sdf file. These libraries were further filtered for any repeated molecule, 3D energy minimized using the Universal Force Field (uff), and exported in sdf format utilizing open-access Open Babel 3.1 software [23].

### 4.3. Molecular Docking

Docking software Gnina version 1.0.2 [24] was used to perform standard molecular docking simulations for each chemical library obtained from MolPort, and reference ligands with their respective protein, considering them as rigid molecules (Table 5). Simulations were carried out considering a cubic grid box centered at the coordinates corresponding to the center of mass for the co-crystallized ligand for each respective protein. The grid box was configured with an edge length of 24 Å for each XYZ dimension, using 1 Å spacing, for a total of 13,824 grid points. The number of poses for each ligand was set to 9 by default, and the most energetically favorable pose for future protein–ligand intermolecular interactions was selected. Docking protocol validation was performed by re-docking from co-crystallized ligands and RMSD calculations.

### 4.4. Molecular Docking Analyses

Docking results were analyzed considering two criteria, BFE and interaction profiles of amino acid residues. Initially, ligands were filtered out based on their BFE value in comparison to the respective reference ligand; only compounds with a higher affinity than the reference ligand were considered for the next step. Then, compounds that satisfy the energy criterion were determined by their interaction profile using Protein-Ligand Interaction Profiler (PLIP) version 2.2.2 software [25]. This profile was considered a fingerprint that was then compared to the fingerprint of the reference ligand. Using the Python 3.14 package sklearn, the Jaccard distance was determined for fingerprints to obtain a distance matrix for each library. Using the Euclidian metrics of the Kmeans clustering function of sklearn on the distance matrix, the distortion and inertia measures were determined and graphed to determine the optimal number of clusters to divide the data into, considering the “elbow method” [26]. Data was clustered in the N number of clusters based on previously determined, and the top ten compounds with the best BFE values grouped with reference ligands were considered for purchase and in vitro studies.

### 4.5. Trypanocidal Assay

CD1 mice, 6 to 8 weeks old, infected with bloodstream trypomastigotes of INC-5 and NINOA strains, were used for the trypanocidal assay. At the peak of parasitemia (4 to 6 weeks), blood was collected by cardiac puncture using sodium heparin as an anticoagulant. Cells were adjusted to 1 × 10^6^ trypomastigotes/mL. In a 96-well plate, 90 µL of infected blood and 10 µL of the selected compounds or dilutions of the reference drugs benznidazole and nifurtimox were deposited for a final volume of 100 µL per well. Reference drugs were used as positive lysis control, and wells with untreated blood trypomastigotes were used as negative lysis control; the microplates were incubated at 4 °C for 24 h. Motile trypomastigotes were subsequently quantified using the well-described Pizzi–Brener method [27], for which 5 μL of blood was placed on a slide and covered with a 13 × 13 mm coverslip. Motile protozoa were counted in 15 fields at 40× using an optical microscope [28]. The compounds were evaluated at five different concentrations using serial dilutions (50–3.125 μg/mL), with the percentage of lysis determined and the LC_50_ calculated using the Probit statistical tool. The assay was performed in triplicate. Finally, the results were converted into micromolar units. The analyzed compounds were purchased from the commercial supplier MolPort and used without further purification. MolPort codes of the compounds are: MolPort-001-909-951 (LK-284), MolPort-042-617-095 (TD-095), MolPort-003-370-655 (TD-655), MolPort-002-260-936 (TS-936), MolPort-001-570-230 (TS-230), MolPort-002-264-967 (TIM-967), MolPort-000-631-831 (TD-831), and MolPort-000-630-149 (TD-149).

### 4.6. Statistical Analysis

All analyses were performed using Microsoft Excel, and results are expressed as mean ± standard deviation. Means were compared using one-way ANOVA and Tukey’s test, as indicated in the table legend. Differences were considered statistically significant when *p* ≤ 0.05.

### 4.7. Molecular Dynamics

MD simulations were performed for chosen compounds to analyze the protein–ligand complex stability and thus assess their potential as stable *Tc*TS and *Tc*TIM inhibitors. The analysis was performed using the GROMACS version 2018.4 software [29] with a 200 ns final simulation in triplicate at a temperature of 300 K [30,31]. The topology of each compound was generated with the ACPYPE Antechamber module using the General Amber Force Field, and the protein force field was Amber99SB-ILDN. The system was solved by adding water molecules in a dodecahedron with a minimum distance from the wall of 10 Å, using the TIP3P water model. After, the system was neutralized by adding Na^+^/Cl^−^ ions and energy-minimized by the steepest descent algorithm (50,000 times). The equilibrium steps were conducted at 300 K in two steps: (1) the ligand was simulated at NVT conditions (constant number of particles, volume, and temperature) using a V-rescale thermostat considering a time constant (tau_t) of 0.1 ps obtaining velocities according to Maxwell–Boltzmann distribution; (2) the ligand was simulated at NPT conditions (constant number of particles, pressure at 1 atm, and temperature) utilizing a V-rescale thermostat and a Berendsen barostat with time constants (tau_t and tau_p) of 0.1 and 2.0 ps, respectively. Each step was achieved at 100 ps. The criteria for RMSD convergence considered a stabilization ≤ 2 Å. The stability of the complex was determined by RMSD, RMSF, and Rgyr calculations using the GROMACS 2025.1 software tools [32,33].

### 4.8. In Silico Pharmacokinetic Analysis

Selected compounds were analyzed using the ADMETlab 3.0 website (https://admetlab3.scbdd.com) [34] (accessed on 20 February 2026) to determine their physicochemical and pharmacokinetic properties, and the ProTox-3.0 server [35] (accessed on 20 February 2026) to predict their toxicity. To accomplish this, the ligand chemical structure was represented in SMILES format. Then, only the properties of interest were chosen for prediction.

## 5. Conclusions

Ligand-based virtual screening of FDA-approved drugs ketanserin, terfenadine, sulfasalazine, doxazosin, glipizide, and glyburide yielded eight new compounds with anti-*T. cruzi* activity superior to that of the reference drugs benznidazole and nifurtimox. These compounds were predicted to form interactions and maintain stability in complex with *Tc*TS and *Tc*TIM throughout MD simulations. Hence, these findings support the idea that trypanocidal activity involves the inhibition of these essential proteins, although further enzymatic assays are needed to confirm. All compounds met the drug-likeness criteria without causing hepatotoxicity, with compound TD-095 standing out as a promising candidate. These results encourage the continued study of FDA-approved drug blocks using CADD to discover new and more potent agents for the pharmacological treatment of Chagas disease.

## Figures and Tables

**Table 1:** Molecular docking analysis of co-crystallized ligands on *trans*-sialidase and triosephosphate isomerase from *T. cruzi*.

Protein (PDB ID)	Reference Inhibitor	BFE (kcal/mol)	Interaction Profile
*Tc*TS (1MS8)	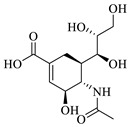 DANA	−8.1	HB: ARG53, ASP96, TRP120, GLN195, GLU230, ARG245, TYR342 SB: ARG30, ARG245, ARG314 WB: TYR119, ARG245
*Tc*TIM (1SUX)	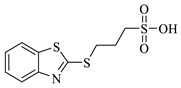 BTS	−6.2	HB: TYR103 HI: TYR102, ILE69, PHE75 π-SI: TYR103

HB: Hydrogen bonds; HI: Hydrophobic interactions; π-SI: π-stacking interactions; SB: Salt bridges; WB: Water bridges.

**Table 2:** Compounds obtained from virtual screening based on FDA-approved drugs from the MolPort database.

Scaffold Used	Criteria						
	Structural Similarity 50%	Lipinski-Veber Rules	BFE *	Interaction Analysis			
				Interactions	Grouping by Interactions	Control-Like Group	Compounds to Acquire
***Trans*-sialidase**							
Ketanserin tartrate	10,000	7747	7040	152	10	5	TD-095
Terfenadine	3943	3296	3290	84	9	8	TS-230 TS-936
Sulfasalazine	10,000	8161	8161	315	10	6	TD-831 TD-149
Doxazosin mesylate	10,000	1397	1397	410	9	7	TD-655
**Triosephosphate isomerase**							
Glipizide	10,000	18,311	18,218	13,612	10	5	LK-284 TIM-967
Glyburide							
Gliquidone							

* Compounds with better BFE that control ligand.

**Table 3:** Half-maximal lytic concentration (LC_50_) of the eight FDA drug analogues selected by virtual screening against *T. cruzi* trypomastigotes.

Scaffold	Derivative	*T. cruzi* (µM)	
		NINOA	INC-5
***Trans*-sialidase**			
Ketanserin tartrate	TD-095	48.60 ± 1.04 ^b^	13.75 ± 0.82 ^bd^
Doxazosin mesylate	TD-655	>200 ^NS^	>200 ^NS^
Terfenadine	TS-936	71.55 ± 3.05 ^b^	37.54 ± 0.64 ^b^
	TS-230	>200 ^NS^	70.86 ± 2.12 ^b^
Sulfasalazine	TD-831	75.94 ± 3.80 ^b^	26.17 ± 1.28 ^bc^
	TD-149	40.54 ± 2.36 **^bc^**	>200 **^NS^**
**Triosephosphate isomerase**			
Glyquidone Gliburide Glipizide	TIM-967	69.70 ± 7.89 ^b^	39.69 ± 2.01 ^b^
	LK-284	116.70 ± 0.90 ^b^	82.29 ± 1.66 ^b^
Benznidazole Nifurtimox		161.05 ± 1.08 **^a^**	254.93 ± 0.38 **^a^**
		219.82 ± 0.58 **^a^**	317.85 ± 0.21 **^a^**

ANOVA: *p* < 0.05; a−d: Comparison of means by Tukey; NS: not significant.

**Table 4:** ADMET prediction of compounds with in vitro activity against *T. cruzi*.

ADMET Properties	TD-095	TS-936	TD-831	TIM-967	LK-284	Benznidazole	Nifurtimox
MW (g/mol) < 500	401.16	474.2	413.09	436.15	436.11	260.09	287.06
RB < 10	4	9	4	8	5	6	3
HBA < 10	9	5	7	6	7	7	8
HBD < 5	0	2	2	1	1	1	0
TPSA (Å^2^) < 140	98.80	61.8	113.68	75.71	84.94	90.06	106.02
LogP < 5	1.15	3.27	3.48	2.69	3.44	0.89	−0.74
LogS −4˜0.5 log mol/L	−2.97	−4.22	−3.81	−3.63	−5.28	−2.86	−3.15
Permeability BBB	No	No	No	Yes	Yes	Yes	No
P-gp substrate	Yes	Yes	Yes	Yes	Yes	Yes	Yes
CYP1A2 inhibitor	Inactive	Inactive	Active	Inactive	Inactive	Inactive	Inactive
CYP2C19 inhibitor	Inactive	Active	Inactive	Active	Active	Active	Inactive
CYP2C9 inhibitor	Inactive	Active	Active	Active	Active	Inactive	Inactive
CYP2D6 inhibitor	Inactive	Inactive	Inactive	Active	Active	Inactive	Inactive
Hepatotoxicity	Inactive	Inactive	Active	Inactive	Active	Inactive	Inactive
Predicted LD_50_ (mg/kg)	1000	800	4000	650	4000	2000	1500

MW: Molecular weight, RB: rotatable bonds, HBA: hydrogen bond acceptors, HBD: hydrogen bond donors, PSA: polar surface area, LogP: partition coefficient, LogS: solubility coefficient, P-gp: P-glycoprotein, Permeability BBB: permeability of the blood–brain barrier.

**Table 5:** Reference protein–ligand complexes used, and their respective docked libraries.

Complex	Docked Libraries
1MS8-DANA	Ketanserin tartrate
	Terfenadine
	Sulfasalazine
	Doxasozin mesylate
1SUX-BTS	Glipizide
	Gliquidone
	Glyburide

## Data Availability

The original contributions presented in this study are included in the article. Further inquiries can be directed to the corresponding authors.

## Abbreviations

*T. cruzi*	*Trypanosoma cruzi*
CADD	Computer-Aided Drug Design
BFE	Binding free energy
MD	Molecular dynamics
RMSD	Root mean square deviation
RMSF	Root mean square fluctuation
Rgyr	Radius of gyration
PDB	Protein Data Bank
TS	*Trans*-sialidase
TIM	Triosephosphate isomerase
